# mTOR-mediated nutrient sensing and oxidative stress pathways regulate autophagy: a key mechanism for traditional Chinese medicine to improve diabetic kidney disease

**DOI:** 10.3389/fphar.2025.1578400

**Published:** 2025-04-23

**Authors:** Liu Li, Junju Zou, Tongyi Zhou, Xiu Liu, Danni Tan, Qin Xiang, Rong Yu

**Affiliations:** ^1^ School of Traditional Chinese Medicine, Hunan University of Chinese Medicine, Changsha, China; ^2^ Hunan Key Laboratory of Traditional Chinese Medicine Prescription and Syndromes Translational Medicine, Hunan University of Chinese Medicine, Changsha, China

**Keywords:** autophagy, diabetic kidney disease, sense of nutrition, oxidative stress, traditional Chinese medicine, mTOR, mechanism

## Abstract

**Context:**

Autophagy plays a pivotal role in the pathogenesis of DKD, and the mechanistic target of rapamycin (mTOR) pathway, which regulates nutrient sensing and oxidative stress responses, is a key regulator of autophagy. Traditional Chinese Medicine (TCM) has garnered attention for its potential to treat DKD by modulating the mTOR signaling pathway, reducing oxidative stress, and restoring autophagic function.

**Objective:**

The objective of this study is to examine how mTOR-mediated regulation of nutrient sensing and oxidative stress impacts autophagy in DKD, and to explore how TCM modulates these pathways to improve the condition.

**Methods:**

A systematic review was conducted using PubMed, Web of Science, Wanfang Data, and China National Knowledge Infrastructure (CNKI), with the search extended to December 2024. The search subject terms included ‘diabetic kidney disease,’ ‘Traditional Chinese Medicine,’ ‘mTOR,’ ‘nutrient sensing,’ and ‘oxidative stress.’ Studies were rigorously screened by two investigators.

**Results:**

This review systematically examines the pathogenesis of mTOR-mediated nutrient sensing dysfunction and oxidative stress in DKD, highlighting their impact on autophagy. It further clarifies how these mechanisms are targeted by Chinese medicine in the treatment of DKD. The review summarizes the potential mechanisms by which TCM, including monomers (e.g., Astragaloside IV), individual botanical drugs (e.g., *Dendrobium nobile* Lindl.), and compound formulations (e.g., Tongluo Digui Decoction), regulate autophagy in DKD through pathways such as AMP-activated protein kinase (AMPK), mTOR, sirtuins (Sirt), and the phosphatidylinositol three kinase (PI3K)/Akt/mTOR signaling pathway. TCM compound formulas share a common foundational framework, with the majority being formulated based on therapeutic principles such as ‘Yiqi’, ‘Yangyin’, ‘Tongluo’, and ‘Huashi’.

**Conclusion:**

TCM shows promise in treating DKD, with unique advantages in modulating key signaling pathways. However, the underlying mechanisms remain complex and warrant further investigation.

## 1 Introduction

Diabetic kidney disease (DKD) is one of the most prevalent microvascular complications in diabetic patients and a leading cause of end-stage renal disease (ESRD) (Młynarska et al., 2024). The hallmark pathological features of DKD include thickening of the glomerular basement membrane, renal tubulointerstitial fibrosis, and proteinuria. The treatment options for advanced DKD remain limited, underscoring the urgent need for novel therapeutic strategies (Zoja et al., 2020). Recent studies have shown that autophagy is impaired in the renal cells of patients with DKD (Han et al., 2023), with dysfunction exacerbated under pathological conditions such as hyperglycemia and oxidative stress (Parmar et al., 2022; Zhang et al., 2024).

Autophagy is a cellular process that degrades cytoplasmic proteins and damaged organelles through lysosomal activity, under the regulation of autophagy-related genes (Atg). There are two main types of autophagy: nonselective autophagy, which indiscriminately isolates and degrades parts of the cytoplasm (including organelles and macromolecular complexes), and selective autophagy, which targets specific, often harmful, substrates for degradation (Vargas et al., 2022). Selective autophagy is initiated by the ubiquitination of cargo, which binds to specific receptor proteins such as p62, TAX1BP1, NDP52, NBR1, optineurin, *etc.*, (Johansen and Lamark, 2020). These receptors facilitate the core autophagy process by interacting with ubiquitin-like proteins (UBLs) from the Atg8/LC3/GABARAP family and by directly interacting with Atg11 (in yeast) or FIP200 (in mammals) (Kirkin and Rogov, 2019). Functionally, various autophagy-related proteins regulate different stages of the autophagic pathway. In mammalian cells, autophagy is primarily controlled by approximately 20 core ATG proteins and is tightly regulated by the mechanistic target of rapamycin (mTOR) signaling pathway (Zhang Y. et al., 2022).

Autophagy is closely associated with the onset and progression of DKD. In DKD, an imbalance between protein synthesis and degradation leads to protein accumulation, which exacerbates glomerular hypertrophy and further renal damage, thereby promoting the development of DKD (Dronavalli et al., 2008). Autophagy plays a crucial role in protein degradation, and defects in autophagy caused by diabetes may significantly contribute to the initiation and progression of DKD. Various renal cells involved in glomerular filtration—such as podocytes, mesangial cells, endothelial cells, and renal tubular epithelial cells—are all closely linked to autophagy. Autophagy in these cells plays a protective role in the kidney and is considered a key target in DKD (Zhang Z. et al., 2022). Among these cells, glomerular podocytes exhibit higher basal autophagy levels (Liu et al., 2017), making them a major focus of research (Audzeyenka et al., 2022). Studies have shown that knockout of podocyte-specific autophagy-related genes (ATG5) in mice rapidly induces proteinuria, suggesting that basal autophagy is essential for maintaining podocyte function (Hartleben et al., 2010; Tang et al., 2020). Additionally, Remah et al. (Yassin et al., 2021) also found that the expression of autophagy markers, including ATG5 and LC3B, was downregulated in peripheral blood monocytes from DKD patients and in kidney tissues of DKD mice. However, some studies suggest that excessive autophagy may have detrimental effects on kidney function. Continuous activation of autophagy may not always provide protective benefits and could worsen podocyte injury (Lenoir et al., 2015; Yu et al., 2018). Therefore, maintaining a balance in autophagy is essential for the proper physiological function of the glomeruli.

Malnutrition and metabolic disorders are significant contributors to DKD progression. mTOR is a protein kinase complex that exists in two main forms: mTORC1 and mTORC2. Of these, mTORC1 is the most critical, regulating cell growth, metabolism, proliferation, and autophagy through downstream effector molecules. mTORC1 plays a central role in nutrient sensing. The development of DKD is closely linked to disturbances in glucose metabolism, which can alter mTORC1 activity and, consequently, affect autophagic processes. mTORC2 primarily regulates cytoskeletal reorganization, cell survival, and metabolic homeostasis. Although Akt signaling plays a critical role in insulin resistance, the role of mTORC2 in the pathological processes of diabetic nephropathy is more indirect, and its activation level is likely weaker compared to mTORC1.

Oxidative stress is another critical factor in the progression of DKD. Hyperglycemia-induced oxidative stress damages renal cells, promoting inflammation and fibrosis. mTOR’s role in oxidative stress regulation is multifaceted. First, oxidative stress can activate mTOR. Reactive oxygen species (ROS) signaling can stimulate mTORC1 activation. In the context of DKD, chronic oxidative stress may lead to the over-activation of mTORC1, which further inhibits autophagy, exacerbating cellular damage. At the same time, mTOR itself can suppress the autophagic response to oxidative stress. mTORC1 inhibits autophagy through downstream targets such as ULK1, reducing the cell’s ability to clear damaged components. As a result, cells become more vulnerable to oxidative damage, and the inability to repair damaged cells via autophagy accelerates the deterioration of DKD.

In recent years, increasing attention has been focused on enhancing autophagy in DKD through modulation of the mTOR-mediated nutrient sensing and oxidative stress pathways (Tang et al., 2021). The inherent complexity of DKD, poses significant limitations to conventional therapeutic strategies reliant on singular molecular targeting. In this context, TCM has emerged as a promising alternative, leveraging its multi-target regulatory networks (e.g., mTOR pathway modulation, oxidative stress mitigation, and autophagic flux restoration) to address systemic imbalances in DKD progression. For example, active metabolites such as berberine and Astragaloside IV have been shown to alleviate glomerular and tubular damage by restoring the balance of the mTOR pathway and enhancing autophagic activity. Given these findings, exploring the mechanisms by which Traditional Chinese Medicine (TCM) regulates the mTOR signaling pathway offers promising potential for the development of new therapeutic strategies for DKD (Xue et al., 2024). This review systematically summarizes research on how TCM can improve autophagy through mTOR-mediated nutrient sensing and oxidative stress pathways. It aims to analyze the therapeutic potential of TCM in DKD and propose new avenues for future treatment.

## 2 The mechanism of autophagy regulated by mTOR-mediated nutrient sensing and oxidative stress in DKD

### 2.1 mTOR-mediated autophagy and DKD

In the context of DKD, mTOR expression has garnered significant attention, largely due to its role in dysregulated autophagy. Specifically, the overactivation of the mTOR signaling pathway, a known negative regulator of autophagy, is observed in podocytes in DKD (Leventhal et al., 2017). Numerous studies have demonstrated elevated mTOR activity in db/db mice, streptozotocin (STZ)-induced diabetic rats, and DKD patients (Sakai et al., 2019; Song et al., 2019). Given that autophagy is essential for maintaining podocyte homeostasis, it has been found that inhibiting the mTOR pathway using rapamycin enhances autophagy and reduces podocyte injury (Xiao et al., 2014). Multi-cohort (CRIC/SMART2D/American Indian) studies established UAdCR as a mechanistic ESKD biomarker; proximal tubule mTOR signaling underlies normoalbuminuric pathology (Sharma et al., 2023).Dysregulated autophagy drives diabetic kidney disease (DKD) progression. Clinical studies show reduced p-mTOR/mTOR in DKD (Xiong et al., 2019), while podocyte mTORC1 hyperactivation in obesity CKD exacerbates renal dysfunction (Iwata et al., 2020).

TCM has been shown to inhibit the mTOR pathway, promote autophagy, protect renal function, and reduce renal fibrosis (Xuan et al., 2021). For instance, Akt and AMPK signaling pathways activate mTOR, inhibiting autophagy. Conversely, activation of mTOR by AMPK and p53 signaling can promote autophagy. The pathogenesis of autophagy dysfunction in DKD is linked to the inhibition of AMPK and SIRT1 and the subsequent activation of mTOR (Ren et al., 2020), as well as the regulation of the PI3K/Akt/mTOR pathways (Tang et al., 2022).

It is important to note that autophagy is not a simple binary process, but rather a dynamic and finely regulated mechanism. The regulation of autophagy in the kidney must strike a balance: insufficient autophagy can lead to the accumulation of harmful substances, while excessive autophagy may impose a detrimental burden on cells. Furthermore, the regulatory effects of TCM on mTOR-related pathways in the context of DKD warrant further exploration and investigation.

### 2.2 Studies on the mechanism of autophagy regulated by the mTOR-mediated nutrient sensing pathway in DKD

**FIGURE 1:**
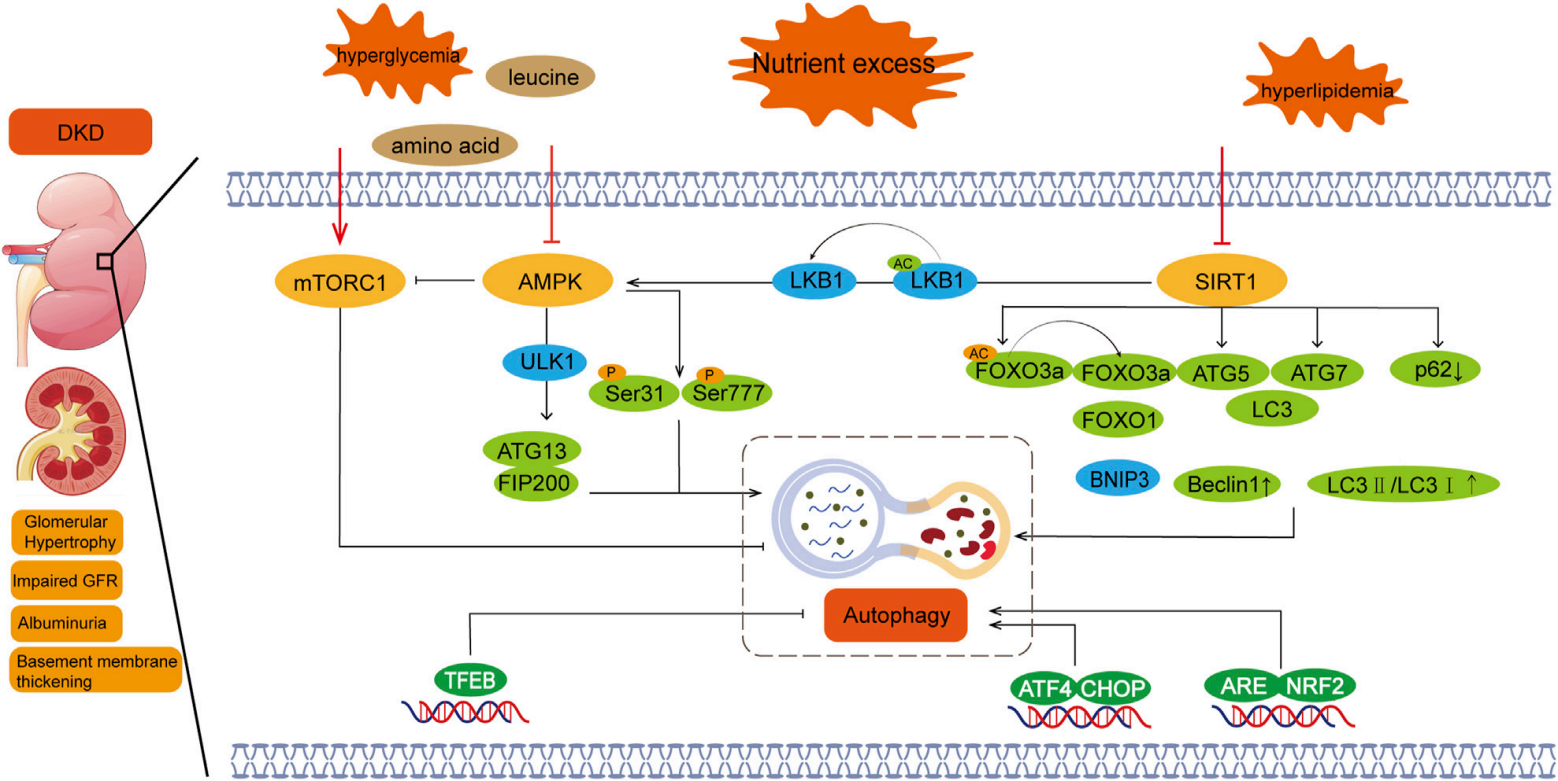
Nutrient sensing pathway and autophagy in diabetic nephropathy. Regulation of autophagy involves three major nutrition-sensing pathways: mTOR, AMPK, and SIRT1 signaling pathways. Excessive nutritional conditions, such as hyperglycemia, activate mTOR and inhibit AMPK and SIRT1. There is also crosstalk between these pathways, with SIRT1 activating AMPK and AMPK inhibiting mTOR. mTORC1:Mechanistic target of rapamycin complex 1; AMPK:Adenosine 5‘-monophosphate (AMP)-activated protein kinase; SIRT1:Silent information regulator 1; DKD:Diabetic kidney disease.

Nutrient sensing pathways, including those involving AMPK, mTOR, and Sirt1, have been widely recognized for their roles in regulating autophagy in diabetic complications (Parmar et al., 2022). In general, proper regulation of AMPK, SIRT1, and mTOR activities—especially enhancing AMPK and SIRT1 function while inhibiting mTOR signaling—could help restore or enhance autophagic activity, potentially mitigating the pathological changes associated with DKD. The crosstalk of the trophic pathways is shown in Figure 1.

mTORC1 regulates autophagy through nutrient-sensing signals, such as amino acids, glucose, and energy status. The development of DKD is closely linked to disturbances in glucose metabolism, which can lead to abnormal activation of mTORC1 and inhibition of autophagy, thereby exacerbating kidney damage (Hinden et al., 2022). In the nutrient sensing pathway, amino acids, particularly leucine, are potent activators of mTORC1 (Zhao et al., 2024a). In diabetic patients, metabolic abnormalities can lead to alterations in amino acid signaling pathways, which, in turn, affect mTORC1 activity and disrupt the autophagy process (Corsetti et al., 2024; Singh et al., 2024). Additionally, HG levels activate mTORC1 through various mechanisms, including the PI3K/Akt signaling pathway. Overactivation of mTORC1 inhibits autophagy, impairing the kidney’s ability to clear accumulated damaged proteins and thus promoting the progression of DKD (Wang H. et al., 2024). From the perspective of energy sensing, AMPK acts as a sensor of cellular energy status. Under low energy conditions, AMPK inhibits mTORC1 activity and activates autophagy to help cells restore energy balance (Deng et al., 2024; Huynh et al., 2023). In DKD, however, AMPK activity may be inhibited, disrupting the regulation of mTORC1 and impairing autophagic function (Oza et al., 2021).

AMPK is an energy-sensing enzyme that plays a pivotal role in initiating autophagy by directly activating ULK1/2 (autophagy-initiating kinases 1/2). Upon activation, AMPK can directly phosphorylate ULK1 or engage in crosstalk with mTOR to induce autophagy (Haq et al., 2019). When mTOR is inhibited by rapamycin, the interaction between ULK1 and AMPK is enhanced, and ULK1 phosphorylation by AMPK increases. This complex crosstalk and feedback between these interconnected proteins fine-tune the autophagic response under metabolic stress conditions (Hou et al., 2023).

In hyperglycemic states, AMPK activity is typically inhibited, which reduces its ability to promote autophagy (Wang et al., 2020). AMPK acts as a central mediator in cellular responses to energy stress and mitochondrial damage, regulating various aspects of autophagy and mitochondrial function (Herzig and Shaw, 2018). As a positive regulator of autophagy, AMPK levels are found to be decreased in DKD. Inhibition of AMPK prevents the ULK1-ATg13-FIP200 complex from inhibiting autophagy, since the ULK1-FIP200 complex is the initiator of autophagy through ULK1 phosphorylation.

In DKD, TCM exerts a renoprotective effect by inhibiting apoptosis and enhancing podocyte autophagy through the AMPK/mTOR signaling pathway (Liu H. et al., 2021). By activating the AMPK/mTOR-mediated autophagy pathway, TCM can reduce mesangial cell proliferation, oxidative stress, inflammation, and ECM accumulation. This regulation of glomerular mesangial cell dysfunction helps alleviate the progression of DKD. Targeting AMPK in TCM represents a promising therapeutic strategy for DKD (Wei et al., 2021). In clinical studies, AMPK signaling has been shown to play a crucial role in improving DKD. To exert its protective effects, AMPK signaling interacts with other molecular pathways, including PGC-1α, PI3K/Akt, NOX4, and NF-κB (Entezari et al., 2022).

Sirt1 is another key regulator in the autophagy process, particularly in metabolic diseases such as DKD (Cantó et al., 2009; Kitada et al., 2017; Wang W. et al., 2019). Sirt1 is a potent positive regulator of autophagy, but its expression is often suppressed in the kidneys of various animal models of diabetes. Sirt1 regulates autophagy-related gene expression by deacetylating the transcription factor FOXO, and increase the expression of BNIP3,thereby promoting autophagy and reducing cell damage in DKD (Han et al., 2023). SIRT1 also increased the levels of Beclin1, ATG5, ATG7 and LC3, increased the ratio of LC3II/LC3I and decreased p62.Additionally, Sirt1 interacts with AMPK and mTOR. The AMPK/Sirt1 axis plays a protective role in DKD by not only restoring autophagic function but also maintaining mitochondrial health (Siddhi et al., 2022). In type 2 diabetic patients, inhibition of Sirt1 and AMPK significantly reduces autophagy levels in glomerular podocytes and renal tubules, which can increase susceptibility to kidney injury. Conversely, under conditions of nutrient deprivation and hypoxia, the AMPK/Sirt1 axis, along with hypoxia-inducible factors (HIF-1α and HIF-2α), drives autophagy and protects the kidney by restoring autophagy, regulating sodium transport, and modulating inflammatory pathways (Jin et al., 2023; Liu H. et al., 2021; Packer, 2020). Therefore, Sirt1 agonists and bromodomain inhibitors may emerge as therapeutic strategies for diabetic nephropathy and could potentially offer new treatment directions for other organ damage caused by diabetes (Zhong et al., 2018).

AMPK/SIRT1/mTOR signaling exhibits stage-specific regulation in DKD. Early-stage compensatory autophagy is maintained through AMPK-mediated ULK1 phosphorylation at Ser317 and SIRT1’s dual deacetylation of LKB1/TSC2, forming a protective loop that suppresses mTORC1 (Yuan et al., 2023). In advanced stages, NAD + depletion inactivates SIRT1, impairing AMPK activation and unleashing mTORC1-driven ULK1 hyperphosphorylation at Ser757, culminating in autophagic failure (Yau et al., 2019). This regulatory shift underlies the pathological transition from adaptive autophagy to proteotoxic stress (Suda et al., 2019).

### 2.3 Study on the mechanism of autophagy regulated by mTOR-mediated oxidative stress pathway in DKD

Oxidative stress refers to cellular damage caused by the accumulation of ROS, while autophagy is a cellular mechanism that helps cells respond to various stressors by degrading and recycling damaged components to maintain survival. The relationship between oxidative stress and autophagy is complex (Sherkhane et al., 2023). Under normal conditions, moderate levels of ROS can promote autophagy to maintain cellular homeostasis. However, in DKD, when cellular damage, such as oxidative stress and protein accumulation, begins to occur, ROS can act as signaling molecules that induce autophagy through oxidative modifications of proteins (Tang et al., 2020). In this context, autophagy serves a protective role by repairing damaged organelles and proteins, thus helping to maintain kidney function and structure (Jaikumkao et al., 2024).

Oxidative stress and DKD are closely linked in the pathogenesis of the disease (Ma F. et al., 2023). In certain stages of DKD or under pathological conditions such as hyperglycemia and hyperlipidemia, excessive ROS production can overwhelm the antioxidant defense capacity of cells, leading to impaired autophagy and extensive damage to cellular structures and functions. However, under conditions of persistent oxidative stress, autophagy can become dysregulated and over-activated, leading to autophagic cell death. Therefore, it is essential to restore autophagic homeostasis by inhibiting both oxidative stress and excessive autophagy under these conditions (Dusabimana et al., 2021; Liu et al., 2025).

**FIGURE 2:**
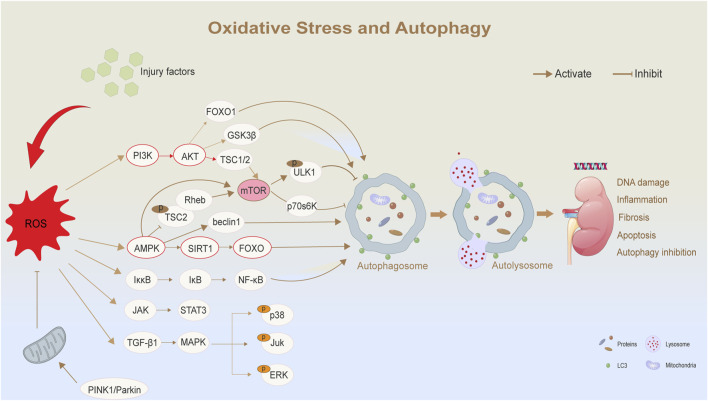
Oxidative stress and autophagy in diabetic nephropathy. ROS regulates autophagy and its related biological effects through a variety of signaling pathways. Elevated levels of ROS are triggered by external injury factors or mitochondrial dysfunction. As a central regulator, ROS affects multiple signaling pathways, such as AMPK/SIRT1/mTOR and PI3K/AKT/mTOR pathways, thereby regulating autophagy. ROS:Reactive Oxygen Species; PI3K:Phosphatidylinositol 3-kinase; LC3:microtubule-associated-proteinlight-chain-3; FOXO:Forkhead box O.

Since mTORC1 plays a central role in cellular metabolism, ROS can influence the overall metabolic state of cells by regulating mTORC1 activity (Yasuda-Yamahara et al., 2021). Therapeutically, the inhibition of mTORC1 activity serves as a primary approach. Studies have demonstrated that mTOR inhibitors, such as rapamycin, can reduce mTORC1 activity, thereby promoting autophagy, reducing oxidative stress and inflammation, and alleviating the progression of DKD (Lin et al., 2021). Second, mTORC1 activity can be modulated indirectly by regulating AMPK and Akt signaling pathways, which further influence autophagy. For example, mTORC1 activity is affected by inhibiting the PI3K/Akt signaling pathway (Kma and Baruah, 2022). Akt, under normal conditions, promotes cell survival and proliferation by activating mTORC1. However, its inhibition leads to an increase in autophagic activity (Liu et al., 2020). Thus, the PI3K/Akt/mTOR axis represents a key pathway for regulating oxidative stress and autophagy. Third, the activation of mTORC1 driven by oxidative stress can be mitigated by antioxidant treatments aimed at reducing ROS generation or enhancing the antioxidant capacity of cells (Zhou et al., 2024). For example, the use of antioxidants such as N-acetyl-L-cysteine and vitamin C has been shown to reduce oxidative stress in DKD (Ma X. et al., 2023). A demonstration of these signaling pathways is shown in Figure 2.

At the same time, hyperglycemia-induced oxidative stress is also recognized as a significant factor contributing to excessive autophagy in DKD. While autophagy initially acts as a self-protective mechanism, severe oxidative stress can result in the overactivation of autophagy, leading to excessive degradation of organelles and eventual cell death. This dysregulated autophagy further exacerbates kidney injury.

### 2.4 Relationship between nutrient sensing and oxidative stress in DKD

In DKD, a complex interplay exists between the nutrient sensing pathway and oxidative stress (Wang M. et al., 2024). The overactivation of the mTOR pathway exacerbates oxidative stress, which in turn disrupts normal nutrient sensing responses by aberrantly activating signaling pathways. This feedback loop further promotes the progression of DKD (Wang and Zhang, 2024). The relationship between metabolic dysregulation and oxidative stress in DKD is intricate, with both processes interacting to drive renal cell damage, inflammation, and fibrosis.

In the context of metabolic dysregulation, the PI3K/Akt/mTOR pathway is often abnormally activated in DKD (Dong et al., 2023; Lai et al., 2023). This excessive activation contributes to renal cell proliferation, fibrosis (e.g., the overproliferation of renal tubulointerstitial cells), and apoptosis. Additionally, it impairs autophagy, resulting in defective cellular clearance and accumulation of damaged proteins and organelles (Kaushal et al., 2020). The activation of the mTOR pathway increases the metabolic burden on cells, further amplifying the inflammatory response and oxidative stress.

In addition, in the context of hyperglycemia and hyperlipidemia, DKD is characterized by a state of nutrient metabolism disorder, which directly contributes to the excessive production of free radicals and ROS. ROS not only causes direct damage to kidney tissues but also activates various signaling pathways, such as the NF-κB and JNK pathways, thereby exacerbating the progression of DKD (Wang Y. et al., 2024). Oxidative stress further damages renal cells, triggers inflammatory responses, activates the mTOR pathway, and inhibits autophagy, leading to aggravated intracellular damage. Interestingly, ROS can also activate signaling molecules such as AMPK, which in turn inhibits mTOR activity. This may represent an adaptive response by cells to mitigate oxidative stress (Yang et al., 2020). However, prolonged high levels of oxidative stress can disrupt the regulation of the PI3K/Akt/mTOR pathway, resulting in metabolic dysfunction and progressive renal impairment.

## 3 TCM regulates autophagy through mTOR-mediated nutrient sensing and oxidative stress pathways to intervene in DKD

**FIGURE 3:**
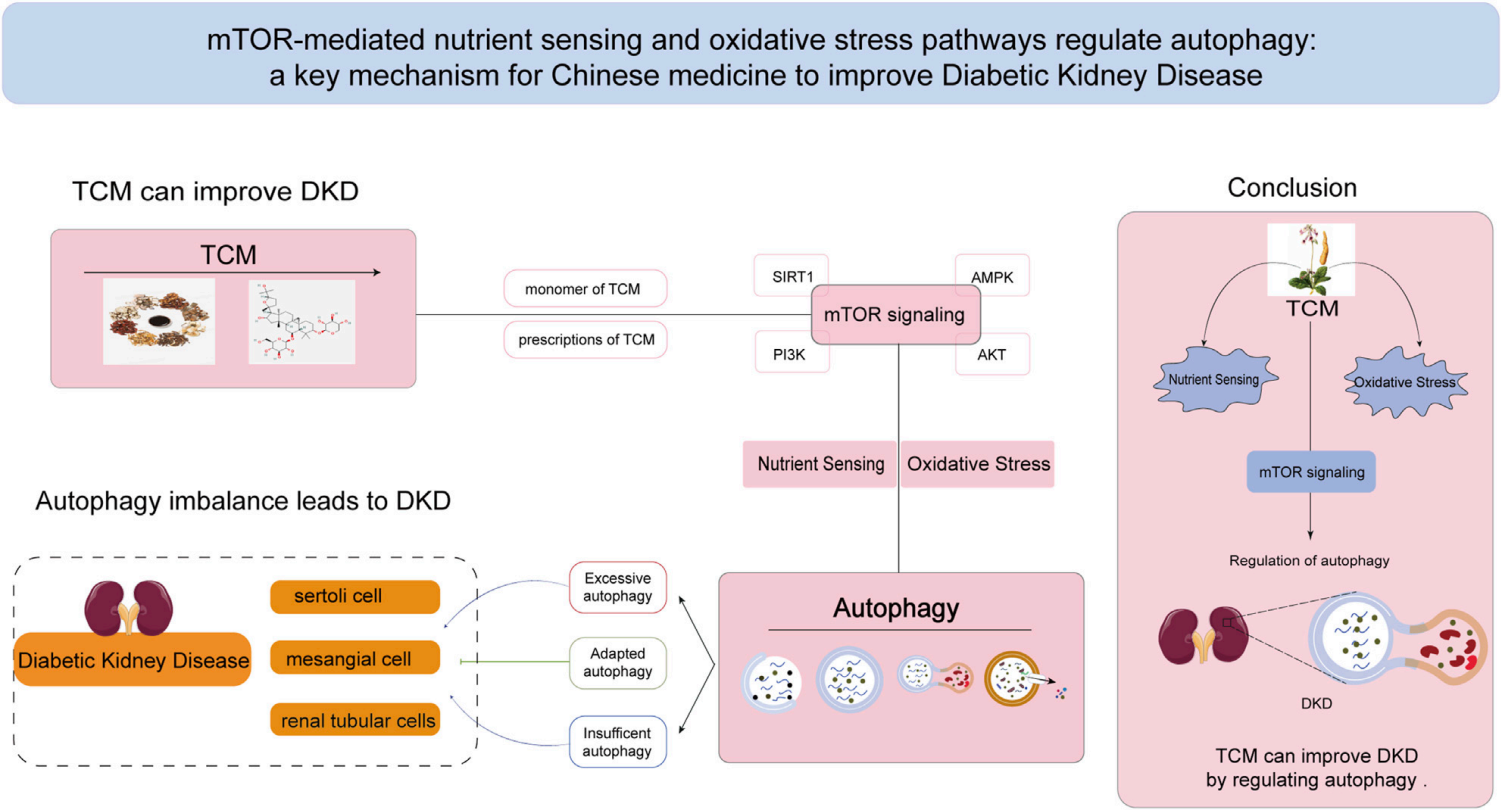
TCM ameliorates DKD by regulating mTOR-mediated nutrient sensing pathway and oxidative stress pathway to affect autophagy. mTOR signaling pathway plays an important role in the regulation of autophagy, and it is also a crucial mechanism by which Chinese medicine affects the balance of autophagy in the treatment of DKD. TCM:Traditional Chinese Medicine.

In the pathological state of DKD, TCM has shown potential in regulating the mTOR pathway to mitigate oxidative stress and reduce cellular damage caused by hyperglycemia. Additionally, TCM can improve nutrient sensing, helping cells adapt to diabetes-related metabolic imbalances. By promoting the activation of autophagy, TCM facilitates the removal of damaged organelles and excess proteins, thereby reducing inflammatory and fibrotic responses in kidney tissue. This review focuses on the role of TCM and its active ingredients in treating DKD by targeting the mTOR pathway to improve oxidative stress and nutrient sensing, ultimately modulating autophagy. These mechanisms are shown schematically in Figure 3.

### 3.1 Single botanical drugs and monomers in DKD that regulate the AMPK/SIRT1/mTOR pathway to mediate autophagy

Numerous TCMs and their active monomers, such as *Rehmannia glutinosa* (Gaertn.) Libosch. ex DC., *Astragalus mongholicus* Bunge, *Anemarrhena asphodeloides* Bunge, *Tripterygium wilfordii* Hook.f., *Coptis chinensis* Franch., and their derivatives, have been shown to modulate the AMPK/SIRT1/mTOR autophagy pathway in DKD. These TCMs are widely applied in clinical practice and have demonstrated efficacy in improving DKD by regulating autophagy.We elaborate on each category based on their distinct classifications.

The terpenoid compounds are as follows:Astragaloside IV, derived from *A. mongholicus* Bunge, is another widely used compound with a significant effect on DKD. Animal studies have demonstrated that it reduces podocyte injury in STZ-induced DKD rats by inducing autophagy via AMPK activation. Notably, this effect is associated with SERCA2-dependent mitigation of endoplasmic reticulum stress (ERS) (Guo et al., 2017). Dioscin, another bioactive compound, ameliorates DKD by inhibiting oxidative stress caused by mitochondria and ERS. It also enhances autophagy and improves mitochondrial quality and quantity control through the AMPK-mTOR pathway (Zhong et al., 2022a). Catalpol belongs to iridoid glycosides. Catalpol, an iridoid glycoside extracted from *R. glutinosa* (Gaertn.) Libosch. ex DC—a commonly used TCM for treating DKD—has been shown to enhance autophagy and lysosomal function by inhibiting mTOR activity and promoting TFEB nuclear translocation. This mechanism stabilizes the podocyte cytoskeleton and restores impaired podocyte autophagy, thereby improving DKD outcomes (Chen et al., 2019).

The flavonoid compounds are as follows:Mangiferin, extracted from *A. asphodeloides* Bunge, has been shown to increase autophagosome formation in podocytes affected by DKD. Its protective effect on podocytes is achieved through upregulation of p-AMPK, downregulation of p-mTOR, and upregulation of p-ULK1 (Wang et al., 2018).Cyanidin-3-O-glucoside (C3G), derived from *Morus alba* L., black rice, and other sources, protects against HG-induced podocyte dysfunction by improving autophagy and reducing cell apoptosis and EMT. These effects are mediated via activation of the SIRT1/AMPK pathway (Wang et al., 2021). Kaempferol (KPF), found in various green plants such as *Brassica oleracea* L., *Corylus avellana* L., propolis, *Citrus × aurantium* f. aurantium, and tea, exerts a protective effect on DKD by reducing apoptosis and enhancing podocyte autophagy. This protective mechanism is associated with the regulation of the AMPK/mTOR pathway (Sheng et al., 2022). Experimental studies have also highlighted the protective effects of Ginkgetin in DKD (Wei et al., 2021). Its mechanism involves reducing HG-induced oxidative stress, inflammation, and extracellular matrix (ECM) deposition via the AMPK/mTOR-mediated autophagy axis. Puerarin, the active compound of *Pueraria montana* var. *lobata* (Willd.) Maesen and S.M.Almeida ex Sanjappa and Predeep, inhibits HG-induced apoptosis. Research has shown (Li et al., 2020) that puerarin promotes AMPK-dependent autophagy by reducing liver kinase B1 (LKB1) acetylation in DKD. Additionally, puerarin can upregulate HMOX1 and Sirt1-mediated autophagy, thereby mitigating diabetes-induced podocyte injury.

Berberine belongs to alkaloids. *Coptis chinensis* Franch., another commonly used TCM in diabetes and DKD, contains berberine, an active compound with multiple therapeutic effects. Berberine enhances AMPK activation and autophagy while inhibiting mTOR activation and reducing podocyte apoptosis caused by HG or AMPK inhibitors (Jin et al., 2017). Notably, the effect of berberine resembles siRNA-mediated mTOR silencing, as it suppresses P70S6k and 4EBP1 phosphorylation (Li et al., 2020). In addition, berberine interferes with the PI3K/Akt/mTOR signaling pathway by targeting VEGFR2, further inhibiting abnormal mesangial cell proliferation (Yang et al., 2024). Berberine is also linked to the PI3K/Akt/AS160/GLUT1 signaling pathway (Ni et al., 2022), highlighting its broad range of effects and its potential as a therapeutic agent for DKD.

Emodin belongs to anthraquinones. Emodin, a natural modulator, has been found to reduce proteinuria and alleviate renal fibrosis. Previous evidence has demonstrated (Liu Y. et al., 2021) that emodin mediates autophagy through the AMPK/mTOR signaling pathway, thereby reducing apoptosis and podocyte injury in DKD rats. *Cyclocarya paliurus* (Batalin) Iljinsk., a botanical drug, contains triterpenic acids that have been shown to prevent kidney injury and apoptosis in DKD by regulating autophagy. Studies have found that these triterpenic acids increase AMPK phosphorylation, reduce the downstream phosphorylation of mTOR, and activate the AMPK-mTOR pathway to modulate autophagy (Zhang L. et al., 2019). Tripterygium glycoside, exerts its bioactivity through synergistic interactions among multiple constituents, including diterpene lactones (e.g., Triptolide), alkaloids, and triterpenes. Tripterygium glycoside, widely studied in both clinical and experimental settings, has demonstrated significant therapeutic effects in DKD. *In vivo* studies have shown that it prevents podocyte injury in DKD mice by upregulating autophagy and downregulating β-arrestin-1 (Zhan et al., 2019). *In vitro* experiments further revealed that Tripterygium glycoside regulates autophagy via the mTOR/Twist1 pathway, inhibiting epithelial-to-mesenchymal transition (EMT) and apoptosis in podocytes (Tao et al., 2021). Jujuboside A belongs to triterpenoid saponins. Jujuboside A, another TCM monomer, inhibits mitochondrial and ERS-mediated oxidative stress and apoptosis. It regulates the CaMKK2-AMPK-p-mTOR and PINK1/Parkin signaling pathways to enhance autophagy and alleviate DKD-related damage (Zhang Y. et al., 2022).

### 3.2 Single botanical drug and monomers in DKD that regulate the PI3K/Akt/mTOR pathway to mediate autophagy

*Dendrobium nobile* Lindl. has demonstrated significant renoprotective effects in DKD. Animal studies have shown that it reduces kidney damage by inhibiting PI3K, Akt, and mTOR phosphorylation while increasing LC3 and Beclin-1 protein and mRNA levels in renal tissues. These effects are attributed to the inhibition of the PI3K/Akt/mTOR signaling pathway and the activation of renal autophagy (Chen et al., 2021).

Ginsenoside Rh1 and paeoniflorin belong to terpenoids, specifically categorized as triterpenoid saponins and iridoid glycosides, respectively. Ginsenoside Rh1 has been reported to improve DKD by reducing inflammation and apoptosis via the AMPK/PI3K/Akt-mediated signaling pathway (Su et al., 2021). Similarly, paeoniflorin (PF) alleviates diabetic kidney injury by restoring autophagy and inhibiting apoptosis through the VEGFR2-mediated PI3K/Akt pathway (Wang et al., 2022). Paeoniflorin has also been shown to improve advanced glycation end products (AGE)-induced mesangial cell damage via the RAGE/mTOR/autophagy pathway (Chen H. et al., 2017). Celastrol, a potential therapeutic agent for DKD, has been shown to achieve podocyte homeostasis and reduce kidney injury through the PI3K/Akt pathway. High doses of celastrol improve renal function, reduce blood glucose, and decrease 24-h urinary albumin in DKD rats. The mechanism involves increasing LC3-II and renin expression while downregulating PI3K, p-Akt, and NF-κB/mTOR mRNA levels (Nie et al., 2020). Geniposide enhances autophagy by activating AMPK and inhibiting Akt via the ULK1-mediated pathway. This reduces proteinuria, podocyte injury, inflammation, and fibrosis, ultimately improving the development and progression of DKD (Dusabimana et al., 2021).

Astilbin, Rutin, Isoorientin, Wogonin and quercetin belong to flavonoids, specifically categorized into dihydroflavonol glycosides, flavonol glycosides, C-glycosylflavones, and methoxyflavones, respectively. Astilbin, a flavonoid compound, has shown protective effects in DKD by reversing high glucose (HG)-induced autophagy inhibition and regulating the PI3K/Akt pathway to reduce autophagy and apoptosis in HK-2 cells (Chen et al., 2018). Rutin, a polyphenolic flavonoid found in plants such as *Ruta graveolens* L., *Acaciella angustissima* (Mill.) Britton and Rose, *A. mongholicus* Bunge, and *Fagopyrum esculentum* Moench, has shown delayed protective effects on DKD. It inhibits HDAC1 via the PI3K/Akt/mTOR pathway to restore autophagy and alleviate endothelial-to-mesenchymal transition (EndMT) in DKD (Dong et al., 2023). Using a proteomic approach, Kong et al. (2023) demonstrated that isoorientin reverses hyperphosphorylation of TSC2 (S939) under HG conditions, stimulating autophagy by inhibiting the PI3K/Akt/TSC2/mTOR pathway.Wogonin targets the PI3K/Akt/NF-κB signaling pathway to regulate autophagy and inflammation. It reduces the expression of pro-inflammatory cytokines, mitigates autophagy dysfunction, and alleviates renal fibrosis and injury in DKD (Lei et al., 2021). Similarly, animal studies have demonstrated (Guo et al., 2024) that quercetin significantly improves liver, spleen, and kidney injuries in Goto-Kakizaki (GK) rats by promoting autophagy through the PI3K/Akt/mTOR pathway.

Tanshinone IIA belongs to diterpenoid quinones. Tanshinone IIA has been found to alleviate DKD by inhibiting the PI3K/Akt/mTOR pathway in podocytes, regulating autophagy, and reducing inflammation (Li et al., 2024).

Curcumin belongs to polyphenols, specifically categorized as a diarylheptanoid within the curcuminoid subclass. Curcumin plays a protective role in DKD by upregulating E-cadherin and LC3 protein expression while downregulating TWIST1, p62, p-mTOR, p-Akt, and PI3K levels. By inhibiting the PI3K/Akt/mTOR pathway, curcumin induces autophagy and reduces podocyte EMT (Tu et al., 2019).

### 3.3 TCM prescriptions regulating mTOR-related pathways to mediate autophagy in DKD

TCM offers significant advantages in the prevention and treatment of DKD, particularly due to its multi-target and multi-pathway therapeutic properties. Among the mechanisms of TCM formulations in treating DKD, mTOR serves as a key target for regulating autophagy. Of the mTOR-related autophagy pathways, the AMPK/SIRT1/mTOR and PI3K/Akt/mTOR pathways are the most extensively studied. Due to the multi-pathway nature of TCM formulas, many formulations have been shown to regulate and improve DKD through crosstalk and interactions between multiple signaling pathways.Details of these formulas are provided in Table 1.

**TABLE 1:** Chinese medicines and monomers for the treatment of DKD via autophagy regulation.

Medicines	Main plant sources	Modeling method	Administration approach	Targeted pathways	References
Astragaloside IV	*Astragalus mongholicus* Bunge	*In vivo*: C57BL/6J mice + STZ (100 mg kg^−1^) 1 week *In vitro*: MPC5 +HG (30 mmol/L)	*In vivo*: 3, 6, and 12 mg/kg/day AS-IV for 8 weeks *In vitro*: 10, 20, 40 or 80 μM AS-IV for 2 h	mTOR/AMPKα	Guo et al. (2017)
Dioscin	*Dioscorea polystachya* Turcz	*In vitro*: SD rats + high fat diet + STZ (35 mg/kg)	*In vivo*: 20 mg/kg for 8 weeks	AMPK-mTOR	Zhong et al. (2022a)
Catalpol	*Rehmannia* *glutinosa* (Gaertn.) Libosch. ex DC	*In vivo*: Male C57BL/6J mice + STZ (170 mg/kg) *In vitro*: MPC5 + HG (40 mmol/L glucose)	*In vivo*: 30, 60, and 120 mg/kg for 8 weeks *In vitro*: 1, 5, and 10 μmol/L for 48 h	mTOR/TFEB Pathway	Chen et al. (2019)
Mangiferin	*Anemarrhena asphodeloides* Bunge	*In vivo*: SD rats + STZ (53 mg/kg) *In vitro*: MPC5 + high glucose (40 mmol/L)	*In vivo*: 12.5, 25, or 50 mg/kg/day for 12 weeks *In vitro*: 5, 10, and 50 μmol/L for 24 h	AMPK-mTOR-ULK1	Wang et al. (2018)
Cyanidin-3-O-glucoside	*Morus nigra* L., black rice	*In vitro*: MPC5 + HG (25 mM)	*In vitro*: 25, 50, and 100 μM for 24 h	SIRT1/AMPK	Wang et al. (2021)
Kaempferol	*Brassica oleracea* L *Corylus avellana* L., *Citrus × aurantium* f. aurantium	*In vivo*: db/db mice	*In vivo*: 50 and 100 mg/kg/day for 12 weeks	AMPK/mTOR	Sheng et al. (2022)
Ginkgetin	*Ginkgo biloba* L	*In vitro*: (HBZY-1) + HG (30 mM)	*In vitro*: 20 μM for 24 h	AMPK/mTOR	Wei et al. (2021)
Berberine	*Coptis chinensis* Franch	*In vitro*: MPC5 + HG	*In vitro*: 2.5 and 5 µM for 24 h	AMPK-mTOR	Jin et al. (2017)
Emodin	*Rheum palmatum* L	*In vivo*: SD rats + unilateral nephrectomy + STZ (35 mg/kg BW twice at 72-h interval)	*In vivo*: 20 and 40 mg/kg for 12 weeks	AMPK/mTOR	Liu et al. (2021b)
The triterpenic acid-enriched fraction of *Cyclocarya paliurus* (Batalin) Iljinsk	*Cyclocarya paliurus* (Batalin) Iljinsk	*In vivo*: SD rats + STZ (65 mg/kg) *In vitro*: HK-2 cell + HG (60 mM)	*In vivo*: 40 and 160 mg/kg for 9 weeks *In vitro*: 5 and 20 μg/mL for 48 h	AMPK/mTOR	Zhang et al. (2019b)
Tripterygium glycoside	*Tripterygium wilfordii* Hook. f	*In vitro*: MPC5 + DKD mice serum (C57BLKS/J db/db) for 24h	*In vitro*: 1.25 μg/mL for 72 h	mTOR/Twist1	Tao et al. (2021)
Jujuboside A	*Ziziphus jujuba* Mill	*In vivo*: SD rats + high-fat diet + STZ (35 mg/kg)	*In vivo*: 20 mg/kg for 8 weeks	CaMKK2-AMPK-p-mTOR	Zhong et al. (2022b)
Rutin	*Ruta graveolens* L.,*Acaciella angustissima* (Mill.) Britton and Rose	*In vivo*: db/db mice *In vitro*: GEnC + HG (30 mM/L)	*In vivo*: 100 and 200 mg/kg/d for 8 weeks *In vitro*: 12.5 μM for 24 h	PI3K/AKT/mTOR	Dong et al. (2023)
Ginsenoside Rh1	*Panax ginseng* C. A. Mey	*In vivo*: C57BL/6 mice + HFD + STZ (100 mg/kg)	*In vivo*: 5 and 10 mg/kg for 8 weeks	AMPK/PI3K/Akt	Su et al. (2021)
Paeoniflorin	*Paeonia lactiflora* Pall	*In vivo*: C57BL/6J mouse + STZ (50 mg/kg)	*In vivo*: 50, 100, and 200 mg/kg for 12 weeks	PI3K-Akt	Wang et al. (2022)
Celastrol	*Tripterygium wilfordii* Hook. f	*In vivo*: SD rats + high fat and high glucose diet + STZ (35 mg/kg)	*In vivo*: 0.5 and 1.5 mg/kg/d for 4 weeks	PI3K/AKT/mTOR	Nie et al. (2020)
Geniposide	*Gardenia jasminoides* J.Ellis	*In vivo*: C57BL/6 male mice + unilateral nephrectomy (UNx) + HFD + STZ (100 mg/kg)	*In vivo*: 50 mg/kg/d for 5 weeks	AMPK/AKT	Dusabimana et al. (2021)
Astilbin	*Astilbe chinensis* (Maxim.) Franch. and Sav	*In vitro*: HK-2 cell + HG (30 mM)	*In vitro*: 10 and 20 μg/mL for 24 h	PI3K/Akt	Chen et al. (2018)
*Isoorientin*	*Parkinsonia aculeata L.,Zea mays*	*In vivo*: C57BL/6J mice + high fat diet + STZ (40 mg/kg) *In vitro*: MPC5 + HG (30 mM)	*In vivo*: 10, 20, and 40 mg/kg for 2 months *In vitro*: 40 μM + 72 h	PI3K-AKT-TSC2-mTOR	Kong et al. (2023)
Wogonin	*Scutellaria baicalensis* Georgi	*In vivo*: SD rats + STZ (30 mg/kg) *In vitro*: HK-2 cell + HG (30 mM)	*In vivo*: 10, 20, and 40 mg/kg for 16 weeks *In vitro*: 2, 4, and 8 μM for 24 h	PI3K/Akt/NF-κB	Lei et al. (2021)
Quercetin	*Quercus infectoria* G.Olivier	*In vivo*: Goto-Kakizaki rats + high-fat diet	*In vivo*: 50 and 75 mg/kg for 10 weeks	PI3K/Akt/mTOR	Guo et al. (2024)
Tanshinone IIA	*Salvia miltiorrhiza* Bunge	*In vivo*: db/db *In vitro*: MPC5 + HG (30 mM)	*In vivo*: 10 mg/kg/day for 12 weeks *In vitro*: 30 μM for 48 h	PI3K/Akt/mTOR	Li et al. (2024)
Curcumin	*Curcuma longa* L	*In vivo*: SD rats + high-fat-sugar diet + STZ (50 mg/kg) *In vitro*: MPC5 + 10% serum from DN rats	*In vivo*: 300 mg/kg for 12 weeks *In vitro*: 40 μM for 24 h	PI3k/Akt/mTOR	(Tu et al., 2019)
Berberine	*Coptis chinensis* Franch	*In vitro*: Mouse podocytes + High glucose (33.3 mM)	*In vitro*: 30, 60, and 90 μM for 24 h	mTOR/P70S6K/4EBP1	Li et al. (2020)
		*In vitro*: MPC5 podocytes + HG	*In vitro*: 2.5 and 5 µM for 24 h	PI3K/AKT/mTOR	Yang et al. (2024)
		*In vitro*: HBZY-1 + AGEs (200 μg/mL)	*In vitro*: 25 and 50 μM for 24 h	RAGE/mTOR	Chen et al. (2017a)
Dendrobium mixture	*Dendrobium nobile* Lindl	*In vivo*: SD rats + high-fat and high-glucose diet + STZ (25 mg/kg)	*In vivo*: 8 g/kg/d for 4 weeks	PI3K/Akt/mTOR	Chen et al. (2021)
Astragalus mongholicus Bunge and Panax notoginseng (Burkill)	—	*In vivo*: C57BL/6 + high-fat and high-sugar diet + STZ (50 mg/kg) *In vitro*: renal mesangial cells + High-glucose (30 mM)	*In vivo*: 1972, 3944, and 7888 mg/kg/d for 8 weeks *In vitro*: 0.5, 2, and 4 mg/ml for 24 h	mTOR/PINK1/Parkin	Wen et al. (2020)
Tang shenning	—	*In vitro*: MPCs + High-glucose (30 mM)	*In vitro*: 1/25, 1/5, and 1 for 24 h	mTORC1 pathway	Xu et al. (2022)
Tongluo Digui decoction	—	*In vivo*: SD rats + STZ (60 mg/kg)	*In vivo*: 5.4, 2.7, and 1.35 g/kg/d for 16 weeks	mTOR phosphorylation	Han et al. (2021)
Qi Di TangShen Granules	—	*In vivo*: db/db	*In vivo*: 3.37 g/kg/d for 12 weeks	AMPK/SIRT1/mTOR	Wang et al. (2019a)
Modified huangfeng decoction	—	*In vivo*: C57BL/6J + 60% high-fat diet + STZ (40 mg/kg) *In vitro*: MPC5 cells + HG (30 mM)	*In vivo*: 50 mg, 1 g, 2 g/kg/d for 6 weeks *In vitro*: 200, 195 μg/ml for 24 h	PI3k/Akt/mTOR	Ni et al. (2025)
Yishen Huashi granule	—	*In vivo*: Male Wistar rats + STZ (30 mg/kg) *In vitro*: CaCO_2_ + LPS (100 μg/mL); HepG_2_ + LPS (1 μg/mL)	*In vivo*: 1.3, 2.7, and 5.4 g/kg/d for 8 weeks *In vitro*: CaCO_2_ + 1 mg/mL; HepG_2_ + 2.5 mg/mL	mTOR/AMPK/PI3K/AKT	Zhao et al. (2023)
Tangningtongluo Tablet	—	*In vivo*: C57BL/6 + STZ (150 mg/kg) *In vitro*: Min6 + palmitate (0.4 mM)	*In vivo*: 1.0 g/kg for 6 weeks *In vitro*: 0.3125 mg/mL for 12 h	PI3K/Akt/mTOR	Ren et al. (2024)
Yiqi Jiedu Huayu Decoction	—	*In vivo*: SD rats + STZ (60 mg/kg)	*In vivo*: 0.6, 1.2, 2.4 g/kg/d for 12 weeks	PI3K/Akt/mTOR	Xuan et al. (2021)
Jiedu Tongluo Baoshen formula	—	*In vivo*: SD rats + HFD + STZ (40 mg/kg)	*In vivo*: 0.5, 1.0, and 2.0 g/kg/d for 8 weeks	PI3K/Akt/mTOR	Jin et al. (2022)
Modified Huangqi Chifeng Decoction	—	*In vivo*: SD rats + Adriamycin (5 mg/kg)	*In vivo*: 12.5 g/kg/d for 6 weeks	PI3K/mTOR	Yu et al., 2018; Zhao et al. (2024b)
Qidan-Dihuang decoction	—	*In vivo*: db/db mice *in vitro* HK-2 and NRK-52E cells + HG (30 mM)	*In vivo*: 1%, 2% in diet *in vitro*: 12.5, 25, 50 mg/L for 24 h	p38MAPK and AKT/mTOR	Kuang et al. (2024)

TCM compound formulas share a common foundational framework, with the majority being formulated based on therapeutic principles such as ‘Yiqi’, ‘Yangyin’, ‘Tongluo’, and ‘Huashi’. Through *in vivo* and *in vitro* experiments, the combination of *A. mongholicus* Bunge and *Panax notoginseng* (Burkill) F.H.Chen has been demonstrated to enhance autophagy by suppressing mTOR and activating the PINK1/Parkin signaling pathway, thereby mitigating inflammatory kidney damage in DKD. (Wen et al., 2020). *In vitro* studies have shown that Tangshenning reduces HG-induced podocyte injury by inhibiting the mTORC1 pathway (including p-mTOR, mTOR, p-p70S6K, p70S6K, ULK1, and 4EBP1) and its downstream targets, while restoring podocyte autophagy (Xu et al., 2022). Tongluo Digui Decoction increases autophagy by inhibiting mTOR phosphorylation, thereby protecting podocytes and slowing the progression of DKD (Han et al., 2021). Wang X. et al. (2019) demonstrated that QiDi TangShen Granules effectively improve DKD by regulating nutrient-sensing molecules involved in autophagy (AMPK, SIRT1, and mTOR). *In vitro* experiments with db/db mice showed that QDTS treatment increased SIRT1 expression and the p-AMPK/AMPK ratio while decreasing the p-mTOR/mTOR ratio. These findings align with previous reports on the active metabolites of QDTS and their effects on nutrient-sensing pathways. Ni et al. (2025) demonstrated through animal experiments that Modified Huangfeng Decoction (MHD) activates podocyte autophagy via the PI3K/AKT/mTOR pathway and regulates gut microbiota and their metabolites, thereby potentially ameliorating diabetic nephropathy. Yishen Huashi Granule improves mitophagy and inhibits the mTOR/AMPK/PI3K/Akt signaling pathway, further protecting against DKD-related kidney damage (Zhao et al., 2023). Using molecular docking, network pharmacology analysis, and animal experiments, Tangningtongluo Tablets have been shown to reduce pancreatic injury in diabetic mice by inhibiting the PI3K/Akt/mTOR signaling pathway and regulating autophagy (Ren et al., 2024).

**FIGURE 4:**
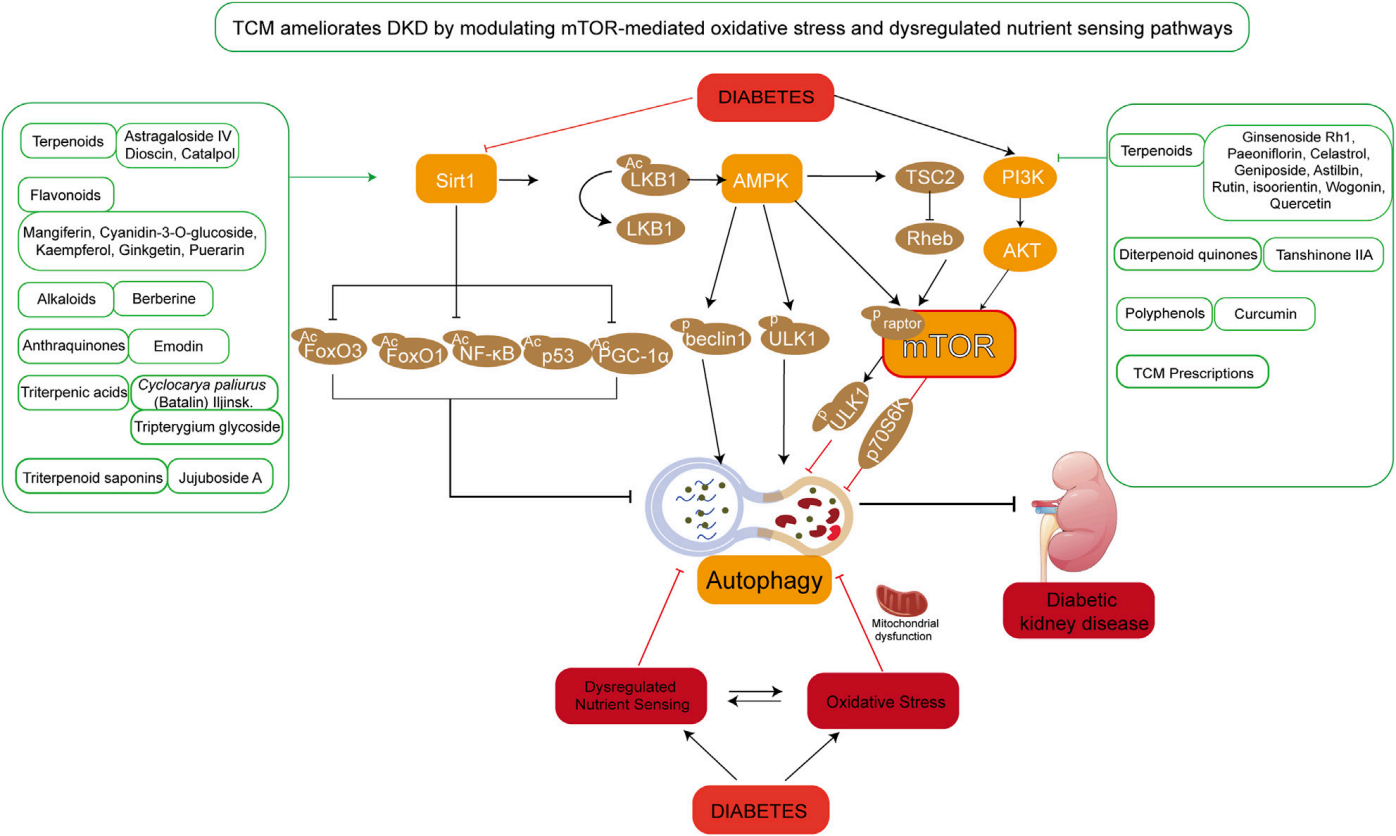
TCM ameliorates DKD by modulating mTOR-mediated oxidative stress and nutrient sensing pathways. Under hyperglycemic conditions, aberrant activation of mTOR-associated signaling pathways leads to dysregulated nutrient sensing and oxidative stress, resulting in mitochondrial dysfunction, suppressed autophagy, and aggravated renal injury. The figure highlights TCM compounds primarily targeting the SIRT1/AMPK/mTOR and PI3K/AKT/mTOR signaling axes. These phytochemicals are categorized into terpenoids, flavonoids, alkaloids, anthraquinones, and other classes.

Animal studies (Xuan et al., 2021) have demonstrated that Yiqi Jiedu Huayu Decoction improves DKD by further inhibiting the mTOR pathway through the regulation of PI3K/Akt and AMPK pathways. This decoction upregulates the expression of autophagy-related proteins LC3-II and Beclin-1 while downregulating p62 expression, thereby promoting autophagy to alleviate podocyte injury, protect renal function, and reduce renal fibrosis. Additionally, it inhibits the PI3K/Akt/mTOR signaling pathway, enhances podocyte autophagy, and reduces proteinuria in DKD. The Jiedu Tongluo Baoshen Formula similarly inhibits the PI3K/Akt/mTOR signaling pathway, enhancing podocyte autophagy and reducing podocyte injury, thus offering an effective treatment for DKD (Jin et al., 2022). Meanwhile, Qidan Dihuang Decoction protects against renal fibrosis in DKD by inhibiting EMT and inflammatory responses via the p38MAPK and AKT/mTOR signaling pathways (Kuang et al., 2024). Interestingly, reducing excessive autophagy is also critical for mitigating kidney injury. Huangqi Chifeng Decoction has been found to activate the PI3K/Akt/mTOR pathway while inhibiting the AMPK/mTOR pathway, exploring the interplay between these two pathways to suppress excessive autophagy and protect the kidneys (Yu et al., 2018; Zhao et al., 2024a). TCMs have shown significant efficacy in the clinical treatment of DKD, and numerous experimental results have provided robust evidence for the role and mechanism of TCM in improving DKD (Zhang L. et al., 2019). These studies offer both a theoretical and scientific foundation for TCM-based therapies for DKD. However, given the complex composition of TCM formulations and the broad range of pathways involved, further research is needed to fully explore their mechanisms and therapeutic potential (The schematic diagram is illustrated in Figure 4).

## 4 Discussion

### 4.1 The unique advantages of TCM

This review highlights the critical role of autophagy regulated by mTOR-mediated nutrient sensing and oxidative stress pathways in the pathogenesis of DKD. It also underscores the therapeutic potential of TCM in treating DKD through these mechanisms. TCM offers distinct advantages in DKD by multi-target regulation, early intervention, and safety. Unlike single-target drugs (e.g., ACEI/ARBs or SGLT-2 inhibitors), TCM formulations holistically improve autophagy, oxidative stress, and metabolic inflammation, achieving albuminuria reversal of early-stage patients and delaying ESRD (Liu et al., 2022; Shen et al., 2024; Wu et al., 2022). TCM also mitigates subjective symptoms (fatigue, edema) unaddressed by conventional therapies and poses lower risks of hypotension/electrolyte disorders, even in advanced CKD (Chen J. et al., 2017). While standardization and large-scale RCTs remain challenges, integrating TCM with emerging strategies (e.g., FDA-aligned quality control) could optimize DKD management through synergistic, patient-centered approaches.

### 4.2 Nutrient-sensing mechanisms: Key players in DKD pathogenesis

While malnutrition is indeed a secondary manifestation of metabolic and renal dysfunction, nutrient sensing dysregulation (e.g., via AMPK/SIRT1/mTORC1 pathways) constitutes a pivotal mechanism in DKD progression, deeply intertwined with oxidative stress and autophagy impairment. These pathways form a triad of pathogenesis: hyperglycemia-induced mitochondrial ROS overproduction suppresses AMPK activity, exacerbating mTORC1-driven anabolic resistance and autophagic flux blockage, which collectively accelerate tubular atrophy and protein-energy wasting.These pathways act as metabolic “hubs,” linking hyperglycemia-induced mitochondrial dysfunction, redox imbalance, and cellular autophagy.

TCM addresses this not by direct nutritional supplementation but by rectifying upstream pathologies: (1) improving tubular energy metabolism via AMPK/SIRT1 to reduce protein catabolism; (2) restoring gut-kidney axis homeostasis to enhance nutrient absorption; (3) mitigating inflammation-driven muscle wasting. This multi-dimensional regulation distinguishes TCM from conventional nutritional therapies, which often neglect underlying disease mechanisms.

### 4.3 Categorization of TCM ingredients by autophagy modulation stages

TCM compounds that regulate autophagy in DKD can be classified into multiple categories: flavonoids, terpenoids, alkaloids, anthraquinones, etc., each demonstrating unique pharmacological characteristics in autophagy modulation. Flavonoids (e.g., astragaloside IV, baicalin) enhance autophagosome initiation and lysosomal fusion by activating AMPK/mTORC1 and upregulating Rab7/LAMP2. Alkaloids like berberine and rhein regulate lysosomal maturation and substrate degradation—berberine elevates lysosomal pH to prolong autophagosome retention, while rhein activates Nrf2-driven mitophagy, reducing renal oxidative damage. Terpenoids (e.g., tanshinone IIA) coordinate autophagy-inflammation crosstalk by inhibiting NF-κB/NLRP3, decreasing IL-1β-induced p62 accumulation in podocytes, while triptolide adaptively regulates AMPK/ULK1 to prevent apoptosis in advanced DKD.

These metabolites classes collectively address DKD’s multifaceted pathogenesis: flavonoids and polysaccharides dominate early-stage interventions by improving autophagic efficiency and metabolic reprogramming, whereas alkaloids and terpenoids target late-stage complications through lysosomal refinement and inflammation resolution.

### 4.4 Existing shortcomings and future research prospects

While TCM shows great promise for DKD management, further research is needed to elucidate its precise mechanisms of action and optimize its clinical application. Future research should delve deeper into the intricate relationship between mTOR and autophagy in DKD and investigate how TCM modulates autophagy and inhibits oxidative stress by targeting upstream signaling molecules of mTOR. Advanced technologies such as high-throughput screening, proteomics, and genomics are essential for comprehensively analyzing the molecular mechanisms of TCM in the treatment of DKD.

Robust clinical trials, particularly randomized controlled trials (RCTs), are needed to evaluate the efficacy and safety of TCM, either as standalone treatments or in combination with modern medicines. Such trials should focus on key efficacy indicators, including renal function, urinary albumin levels, and blood glucose control, while also assessing safety profiles. Existing clinical studies are frequently limited by methodological shortcomings, including small sample sizes, non-standardized TCM formulations, and inadequate blinding protocols. These limitations may exaggerate placebo effects or obscure true treatment outcomes.This direction warrants prioritized attention from the research community.

Furthermore, studies should investigate the therapeutic effects of TCM across different stages of DKD. Given the multi-component nature of TCM, studies on dose optimization and drug interactions are vital. Future research should conduct dose-escalation studies to identify the optimal dose ranges for TCM ingredients, minimizing adverse reactions and interactions with conventional antidiabetic drugs. This approach will enhance both the safety and efficacy of TCM in clinical settings. Patients with DKD exhibit considerable individual variability, necessitating personalized treatment strategies. Combining cutting-edge technologies, such as genomics and metabolomics, with TCM could enable the development of tailored therapeutic approaches. These strategies could address variations in genotypes or clinical subtypes, providing more precise and effective treatments for DKD patients. In conclusion, integrating TCM with modern medical treatments may offer innovative approaches for the comprehensive management of DKD.

Emerging evidence highlights mTOR as a pivotal yet underexplored target in DKD. Conventional mTOR inhibitors (e.g., everolimus) show promise in preclinical models but require rigorous clinical validation for renal safety and efficacy. Meanwhile, TCM-derived metabolites like Astragaloside IV and rhein offer multi-pathway modulation, indirectly targeting mTOR while mitigating oxidative stress and inflammation. Future studies should prioritize TCM-based mTOR inhibitors with refined pharmacokinetic profiles to bridge traditional knowledge and modern pharmacology.

## 5 Conclusion

In the pathological process of DKD, the mTOR signaling pathway plays a critical role in regulating autophagy and maintaining the balance of intracellular metabolism and oxidative stress, thereby mitigating kidney injury. Particularly under conditions of excessive oxidative stress or metabolic dysregulation, the activation or inhibition of the mTOR pathway becomes crucial for the regulation of autophagy, significantly influencing the prevention and treatment of DKD. This review highlights the importance of mTOR-mediated nutrient sensing and oxidative stress pathways in autophagy regulation by analyzing extensive literature. It also emphasizes the potential mechanisms by which TCM improves DKD through these pathways. TCM enhances autophagy in DKD and slows disease progression by modulating mTOR-mediated signaling pathways, particularly the AMPK/SIRT1/mTOR and PI3K/Akt/mTOR pathways. The regulation of autophagy via mTOR-mediated nutrient sensing and oxidative stress pathways represents a key mechanism of TCM intervention in DKD treatment. This underscores the unique advantages of TCM in both the prevention and treatment of DKD, offering promising therapeutic strategies for the effective management of this disease.
